# The predictive value of vessels encapsulating tumor clusters in treatment optimization for recurrent early‐stage hepatocellular carcinoma

**DOI:** 10.1002/cam4.4102

**Published:** 2021-07-01

**Authors:** Zhi‐Yuan Chen, Zhi‐Xing Guo, Liang‐He Lu, Jie Mei, Wen‐Ping Lin, Shao‐Hua Li, Wei Wei, Rong‐Ping Guo

**Affiliations:** ^1^ Department of Gastroenterology Hunan Provincial People’ Hospital Changsha China; ^2^ The First Affiliated Hospital of Human Normal University Changsha China; ^3^ State Key Laboratory of Oncology in South China Guangzhou China; ^4^ Department of Ultrasound Sun Yat‐sen University Cancer Center Guangzhou China; ^5^ Collaborative Innovation Center for Cancer Medicine Guangzhou China; ^6^ Department of Liver Surgery Sun Yat‐sen University Cancer Center Guangzhou China

**Keywords:** hepatocellular carcinoma, radiofrequency ablation, repeat hepatic resection, vessels encapsulating tumor cluster

## Abstract

**Background:**

The predictive value of vessels encapsulating tumor clusters (VETC) in recurrent early‐stage hepatocellular carcinoma (HCC) remains unclear. Therefore, the aim of the present study was to investigate the prognostic significance of VETC in patients with recurrent early‐stage HCC after repeat hepatic resection (RHR) or radiofrequency ablation (RFA).

**Methods:**

From December 2005 to December 2016, 138 patients receiving RHR and 188 patients receiving RFA were recruited. VETC was evaluated by immunohistochemical staining for CD34. The survival outcomes of patients with VETC pattern or not were investigated.

**Results:**

There was no significant difference between the RHR and RFA groups in disease‐free survival (DFS) or overall survival (OS) as determined by the univariate analysis of the whole cohort. In the subgroup analysis of the VETC‐positive cohort, the patients in the RHR group showed a longer median DFS time in contrast to those in the RFA group (15.0 vs. 5.0 months, *p *= 0.001). Similarly, the patients in the RHR group showed a longer median OS time in contrast to those in the RFA group (39.5 vs. 19 months, *p *= 0.001). In the VETC‐negative cohort, no significant differences in DFS and OS rates between the RHR and RFA groups were observed (*p *> 0.05).

**Conclusions:**

The results of our study suggested that RHR was relatively safe and superior to RFA in improving survival outcomes for recurrent early‐stage HCC after initial hepatectomy. Furthermore, the VETC pattern may represent a reliable marker for selecting HCC patients who may benefit from RHR.

## Graphical abstract

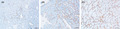

## 1 BACKGROUND

Hepatocellular carcinoma (HCC) is characterized by high vascularization, rapid tumor progression, and extremely poor outcome.^1^, ^2^, ^3^ Surgical resection is commonly accepted as a curative treatment for HCC; however, the long‐term outcomes are not yet satisfactory, as approximately 70% of patients experience recurrence within 5 years.^4^ Approximately 30% of patients with recurrent HCC are diagnosed in the early stage and consequently, bear relatively favorable prognosis.^5^, ^6^ However, guidelines for the management of recurrent early‐stage HCC remain controversial and poorly defined.

Available management options for recurrent early‐stage HCC are almost identical to those for primary HCC. Repeat hepatic resection (RHR) continues to be the conventional option for recurrent early‐stage HCC with preserved liver function and residual liver volume, and progresses in surgical techniques have contributed to enhancing the safety of RHR.^4^, ^7^, ^8^ In addition, radiofrequency ablation (RFA), a minimally invasive option, has emerged as another alternative treatment modality for early‐stage HCC.^9^, ^10^ Several studies have previously recommended RHR when possible in the treatment of recurrent HCC.^11^, ^12^, ^13^ However, conflicting data have shown that RFA, with relatively few complications, could achieve survival outcomes comparable to those of RHR for recurrent early‐stage HCC.^14^, ^15^, ^16^ Therefore, the optimal strategies for recurrent HCC remain unclear and controversial.

We and other researchers have identified two microvessel types in HCC by their distinct morphologic features: capillary‐like with small, scattered capillaries having no or narrow lumen, and sinusoid‐like that form a cobweb‐like pattern and encapsulate tumor clusters, also named vessels encapsulating tumor clusters (VETC).^17^, ^18^ The VETC pattern was found to be consistently and easily detectable in HCC and to indicate a poorer prognosis in patients after recurrence.^19^, ^20^ Moreover, the VETC pattern is acknowledged to be a predictor of sorafenib benefit in patients with HCC, especially those with VETC. VETC can be an indicator for guiding the treatment of patients with recurrent HCC.^21^ However, there are no studies regarding the prognostic value of the VETC pattern in recurrent early‐stage HCC.

In this study, we used tissue specimens containing 326 HCC samples from our center to compare the survival outcomes of recurrent early‐stage HCC treated by RHR or RFA and explore whether VETC pattern could guide the identification of appropriate candidates for optimization treatment.

## 2 METHODS

### 2.1 Patients

From December 2005 to December 2016, a total of 1206 HCC patients developed intrahepatic recurrence after initial R0 resection. Recurrence was diagnosed either by histologic findings or by the noninvasive criteria used by the American Association for the Study of Liver Diseases. Among the patients, 144 (11.9%) were amenable to RHR and 203 (16.8%) received RFA. The inclusion criteria were as follows: (a) age between 18 and 75 years; (b) first intrahepatic recurrence of HCC after curative hepatectomy; (c) single lesion ≤3 cm or 2–3 lesions ≤3 cm recurrent HCC; (d) no radiologic evidence of macroscopic vascular invasion or extrahepatic metastasis; (e) RHR or RFA performed as the initial treatment for recurrent HCC; and (f) Child‐Pugh class A or B. Thus, six patients in the RHR cohort were excluded, namely, four patients who received palliative repeated resection and two patients with other malignancies. Fifteen patients in the RFA cohort were excluded, including five patients older than 75 years, four patients with extrahepatic metastasis, and six patients whose samples were not sufficient for immunohistochemistry staining. Finally, 326 patients were recruited into the current study, including 138 patients receiving RHR and 188 patients receiving RFA as the first treatment for recurrent early‐stage HCC. The study got the approval of the Institutional Review Board of our center and was conducted in line with the ethical guidelines of the Declaration of Helsinki.

### 2.2 Treatment strategy

Treatment for recurrent HCC at the early stage was performed as previously described.^22^ RHR was assigned when there was the possibility for the complete removal of all tumors while retaining a sufficient liver remnant, with an expected remnant liver volume of no less than 250 ml/m^2^, as evaluated by our multidisciplinary team. Resection was avoided if patients had gross ascites, severe portal hypertension, or an inadequate liver remnant. Reasons for assigning RFA instead of RHR included psychological resistance to invasive treatment, refusal of general anesthesia, and an insufficient liver remnant.

### 2.3 RHR procedure

RHR was conducted using the techniques previously described.^22^ The tumor burden, the liver remnant, and the possibility of a negative resection margin were evaluated by intraoperative ultrasonography. If necessary, the Pringle’s maneuver was performed and the clamp/unclamp time of 10 min/5min. Anatomic resection was the preferred surgical method. Nonanatomic resection was performed in the absence of sufficient liver remnant, with a negative resection margin.

### 2.4 Immunohistochemistry staining

The sensitive streptavidin‐biotinylated horseradish peroxidase complex system (Catalyzed Signal Amplification System, DAKO, Carpinteria, CA) was utilized for the immunohistochemistry staining for CD34 based on the manufacturer’s instructions. Formalin‐fixed and paraffin‐embedded sections were dewaxed in xylene and rehydrated in gradient ethanol. Afterward, endogenous peroxidase activity was blocked by 3% hydrogen peroxide for 10 min. Antigen retrieval was performed by microwave pretreatment in 100 W citrate buffers for 5 min and 30 W for 25 min. Then, the sections were incubated with mouse anti‐human CD34 monoclonal antibody (mAb; working dilution 1:200, QBEnd10, DAKO) at 4℃ overnight. Following washing by TBS with 0.1% Tween 20, the sections were incubated with biotinylated rabbit anti‐mouse secondary antibody for 30 min, followed by TBS washing. The sections were then incubated with streptavidin–biotin complex for 15 min. All the sections were counterstained with hematoxylin.

### 2.5 Outcome measures

Immunohistochemical staining was independently evaluated using the same diagnostic criteria by two pathologists who were blinded to the patient data. In case of discrepancy, the specimens were referred to a third observer and the majority decision was considered final. Using a Leica inverted research microscope (Leica Microsystems, Wetzlar, Germany), the slides were examined under 100× magnification to identify the highest vascular density area within the tumor and five representative fields were observed at a higher magnification of 200×. Microvascular invasion (MVI) is defined as microscopic tumor invasion in the central hepatic vein, the portal, or large capsular vessels.^23^, ^24^ Tumor differentiation was histologically graded according to the criteria proposed by the WHO classification of Tumors of the Digestive System (2010 version).

### 2.6 Statistical analysis

Disease‐free survival (DFS) and overall survival (OS) rates were calculated with the life table method, and the survival time was calculated from the day of surgery to the day of death or the most recent follow‐up visit. The patient survival curves were compared using the Kaplan–Meier method and analyzed using the log‐rank test. Chi‐square tests and Spearman’s rank tests were adopted to evaluate the univariate correlation between the biological parameters and clinicopathological variables, as well as the recurrence. The relevant prognostic factors were identified using multivariate Cox proportional hazards models. Statistical Package for the Social Sciences software (SPSS version 19.0, IBM, Armonk, NY, USA) was utilized for data analysis. The *p*‐value was obtained from a two‐tailed test and *p* < 0.05 meant a statistical difference.

## 3 RESULTS

### 3.1 Subject characteristics

**FIGURE 1:**
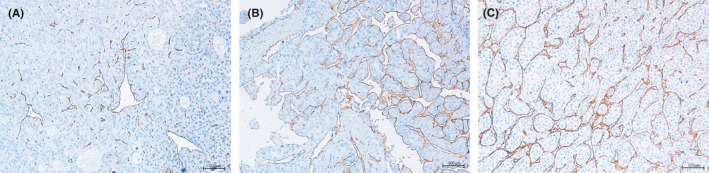
IHC staining for human CD34 was performed to detect vascular patterns in human HCC tissues. (A) VETC‐negative: capillary‐like microvessel; (B) VETC‐positive: sinusoid‐like that form a cobweb‐like pattern and encapsulate tumor clusters; (C) mixed VETC and capillary‐like microvessel. IHC, immunohistochemical staining; HCC, hepatocellular carcinoma; VETC, vessels encapsulating tumor clusters.

**FIGURE 2:**
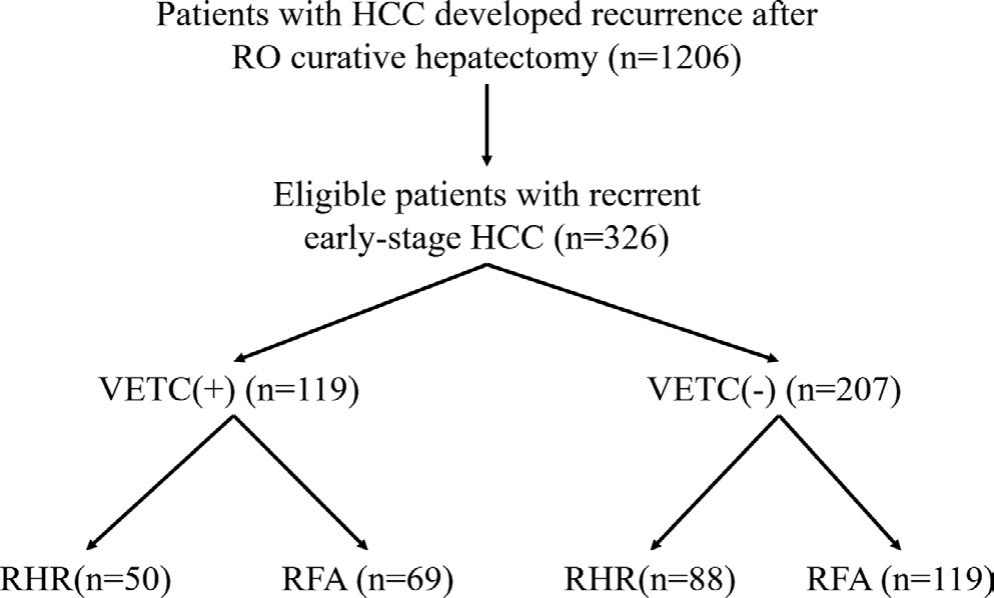
Flow chart of the study protocol. RHR, repeat hepatic resection; RFA, radiofrequency ablation

The baseline characteristics of patients with recurrent early‐stage HCC are summarized in Table 1. In all, 80 patients were VETC‐positive and 145 patients were VETC‐negative. We also found 101 patients with mixed VETC and capillary‐like microvessels in the tumor tissue simultaneously (Figure 1). The value of 55% was the optimal cut‐off value of the VETC phenotype to predict prognosis.^25^ Last, 119 patients were classified as VETC‐positive (defined as the staining of VETC ≥55%) and 207 patients were classified as VETC‐negative (defined as the staining of capillary‐like microvessels ≥55%) (Figure 2). No significant correlation was found with regard to other clinicopathological factors except MVI (*p *< 0.001) (Table S1).

**TABLE 1:** Patient characteristics at the time of recurrence

	VETC (+)			VETC (−)		
Variables	RHR	RFA	*p*	RHR	RFA	*p*
Age, y	49.5 ± 10.9	48.9 ± 10.7	0.822	51.5 ± 10.3	49.4 ± 11.2	0.498
Sex			1.000			0.647
Male	47	64		78	108	
Female	3	5		10	11	
HBsAg			0.427			0.201
Positive	41	61		76	94	
Negative	9	8		12	25	
Background liver			0.708			0.137
Normal	19	29		34	34	
Cirrhosis	31	40		54	85	
Histological grade			0.850			0.776
Well differentiated	21	27		38	48	
Poorly differentiated	29	42		50	71	
Microvascular invasion			0.136			0.472
Present	18	35		14	24	
Absent	32	34		74	95	
ALB at recurrence, g/l	41.9 ± 6.6	42.0 ± 3.3	0.154	42.6 ± 5.8	42.4 ± 7.7	0.488
TBIL at recurrence, umol/l	15.5 ± 4.7	14.7 ± 4.9	0.922	15.5 ± 5.6	15.2 ± 5.9	0.147
HGB at recurrence, g/l	143.6 ± 17.8	143.9 ± 16.8	0.421	144.3 ± 22.4	142.7 ± 24.0	0.777
AFP at recurrence, ng/ml			0.838			0.884
> 20	35	50		56	77	
≤ 20	15	19		32	42	
Child‐Pugh score at recurrence			0.488			0.363
5	42	54		73	94	
6	8	15		13	25	
Tumor size at recurrence, cm	2.4 ± 0.6	2.2 ± 0.5	0.472	2.4 ± 0.5	2.3 ± 0.4	0.630
Tumor multiplicity at recurrence			0.451			0.158
Solitary	44	57		75	91	
Multiple	6	12		13	28	
Time to recurrence			0.192			1.000
< 1 year	23	27		34	54	
≥ 1 year	41	28		46	73	
ECOG‐PS			0.709			0.792
0–1	53	59		93	99	
2	4	3		8	7	

Abbreviations: AFP, α‐fetoprotein; ALB, albumin; ECOG‐PS, Eastern Cooperative Oncology Group Performance Status; HGB, hemoglobin; RFA, radiofrequency ablation; RHR, repeat hepatic resection; TBIL, total bilirubin; VETC, vessels encapsulating tumor clusters

Among VETC‐positive HCC patients, 50 patients underwent RHR and 69 patients underwent RFA; among VETC‐negative HCC patients, 88 patients received RHR and 119 patients received RFA. The proportions of VETC cases were similar between the RHR and RFA groups (42.0 vs. 42.5%). Notably, the VETC pattern was more frequently observed with the occurrence of MVI and early recurrence. In the subgroups of patients with VETC‐positive or VETC‐negative lesions, little significant difference could be observed between the RHR and RFA groups for any of the baseline characteristics.

### 3.2 Survival analysis in the overall cohort

The median duration of follow‐up was 39 months (range, 2–118 months). At the time of censoring, 256 (78.5%) of 326 patients had recurred, and 153 (46.9%) had died of tumor progression. The 1‐, 3‐, and 5‐year DFS rates for the VETC‐positive group and VETC‐negative group were 39.5%, 17.6%, and 8.4% and 50.7%, 29.5%, and 18.8%, respectively. The VETC‐negative group had a longer median DFS time compared with the VETC‐positive group (6.9 vs. 12.0 months, *p *= 0.011). Similarly, the 1‐, 3‐, and 5‐year OS rates for the VETC‐positive group and VETC‐negative group were 74.8%, 44.5%, and 19.3% and 87.4%, 58.9%, and 33.8%, respectively. The VETC‐negative group had a longer median OS time compared with the VETC‐positive group (27 vs. 46 months, *p *= 0.002) (Table 2).

**TABLE 2:** Comparison of median DFS and OS time in entire series (Kaplan–Meier method)

Variables	*n*	DFS	*p*	OS	*p*
HBsAg			0.935		0.932
Positive	272	10.0 ± 0.9		38.5 ± 13.2	
Negative	54	14.1 ± 4.8		39.0 ± 16.2	
Background liver			0.609		0.674
Normal	116	11.0 ± 1.4		39 ± 13.3	
Cirrhosis	210	10.0 ± 1.6		39.0 ± 14.8	
Histological grade			0.028		0.648
Well differentiated	192	10.8 ± 2.2		39.0 ± 13.7	
Poorly differentiated	134	10.0 ± 1.3		38.5 ± 14.4	
Microvascular invasion			<0.001		0.002
Present	90	13.8 ± 1.8		46.0 ± 14.1	
Absent	236	5.7 ± 0.9		27.0 ± 13.5	
AFP at recurrence, ng/ml			0.204		0.123
> 20	108	11.0 ± 3.4		43.5 ± 14.6	
≤ 20	218	10.0 ± 1.2		36.5 ± 9.4	
Child‐Pugh score at recurrence			0.148		0.195
5	146	11.0 ± 1.5		34.5 ± 11.5	
6	180	10.0 ± 1.6		44.0 ± 13.5	
Tumor multiplicity at recurrence			0.094		0.686
Solitary	267	11 ± 1.5		39 ± 17.9	
Multiple	59	7 ± 2.3		39 ± 14.3	
Time to recurrence			<0.001		<0.001
< 1 year	144	7.0 ± 0.9		33 ± 8.3	
≥ 1 year	182	18.0 ± 2.7		50.5 ± 15.0	
VETC			0.011		0.002
Positive	119	6.9 ± 1.4		27.0 ± 10.1	
Negative	207	12.0 ± 1.8		46.0 ± 13.5	
Treatment allocation			0.114		0.259
RHR	138	11.8 ± 3.3		42.0 ± 12.6	
RFA	188	10.0 ± 1.1		37.5 ± 10.4	

Abbreviations: AFP, α‐fetoprotein; DFS, disease‐free survival; OS, overall survival; RFA, radiofrequency ablation; RHR, repeat hepatic resection; VETC, vessels encapsulating tumor clusters.

Univariate analysis exhibited that time to recurrence, VETC, and MVI were found to be significant risk factors affecting DFS and OS. There was little significant difference between the RHR and RFA groups for DFS and OS. By multivariate analysis, time to recurrence (*p *< 0.001), tumor multiplicity (*p *= 0.008), Child‐Pugh score at recurrence (*p *< 0.001), MVI (*p *< 0.001), and VETC pattern (*p *= 0.015) were recognized as independent predictors of DFS. Time to recurrence (*p *= 0.002), Child‐Pugh score at recurrence (*p *= 0.004), MVI (*p *= 0.019), and VETC pattern (*p *= 0.025) were identified as independent predictors of OS (Table 3).

**TABLE 3:** Multivariate analysis of the risk factors related to DFS and OS of recurrent early‐stage HCC by using Cox proportional hazards models

	DFS			OS		
	*p*	HR	95% CI	*p*	HR	95% CI
HBsAg (positive/negative)	0.912	1.019	0.725–1.433	0.779	1.065	0.685–1.656
Cirrhosis (present/absent)	0.627	1.067	0.719–1.220	0.326	1.186	0.844–1.667
Tumor multiplicity at recurrence (solitary/multiple)	0.008	1.554	1.124–2.148	0.514	1.150	0.755–1.752
AFP at recurrence, ng/mL (≤ 20/> 20)	0.461	1.107	0.845–1.450	0.193	1.262	0.889–1.793
Child‐Pugh score at recurrence (5/6)	0.000	2.146	0.338–0.641	0.004	1.848	0.357–0.820
Histological grade (well/poorly differentiated)	0.292	1.147	0.889–1.479	0.354	1.170	0.614–1.191
Time to recurrence (< 1 year/≥ 1year)	0.000	1.799	0.431–0.716	0.002	1.664	0.435–0.832
Microvascular invasion (present/absent)	0.000	2.997	2.103–4.273	0.019	1.739	1.097–2.757
Treatment allocation (RHR/RFA)	0.228	1.170	0.907–1.510	0.459	1.133	0.815–1.575
VETC (positive/negative)	0.015	1.454	0.854–1.948	0.025	1.486	1.050–2.102

Abbreviations: AFP, α‐fetoprotein; DFS, *disease*‐free survival; OS, overall *survival*; RFA, radiofrequency ablation; RHR, repeat hepatic resection; VETC, vessels encapsulating tumor clusters.

**FIGURE 3:**
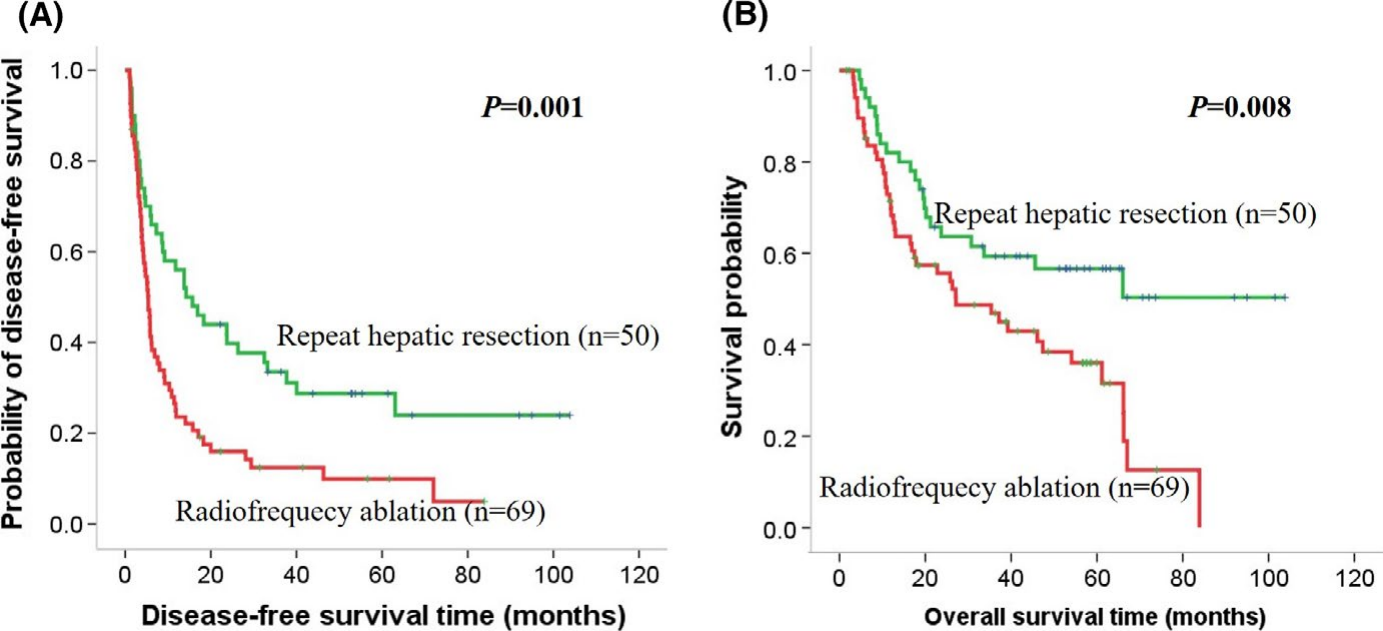
Kaplan–Meier curves of survival outcomes of recurrent early‐stage HCC. Kaplan–Meier curves of (A) DFS and (B) OS in the VETC‐positive cohort. DFS, disease‐free survival; OS, overall survival

**FIGURE 4:**
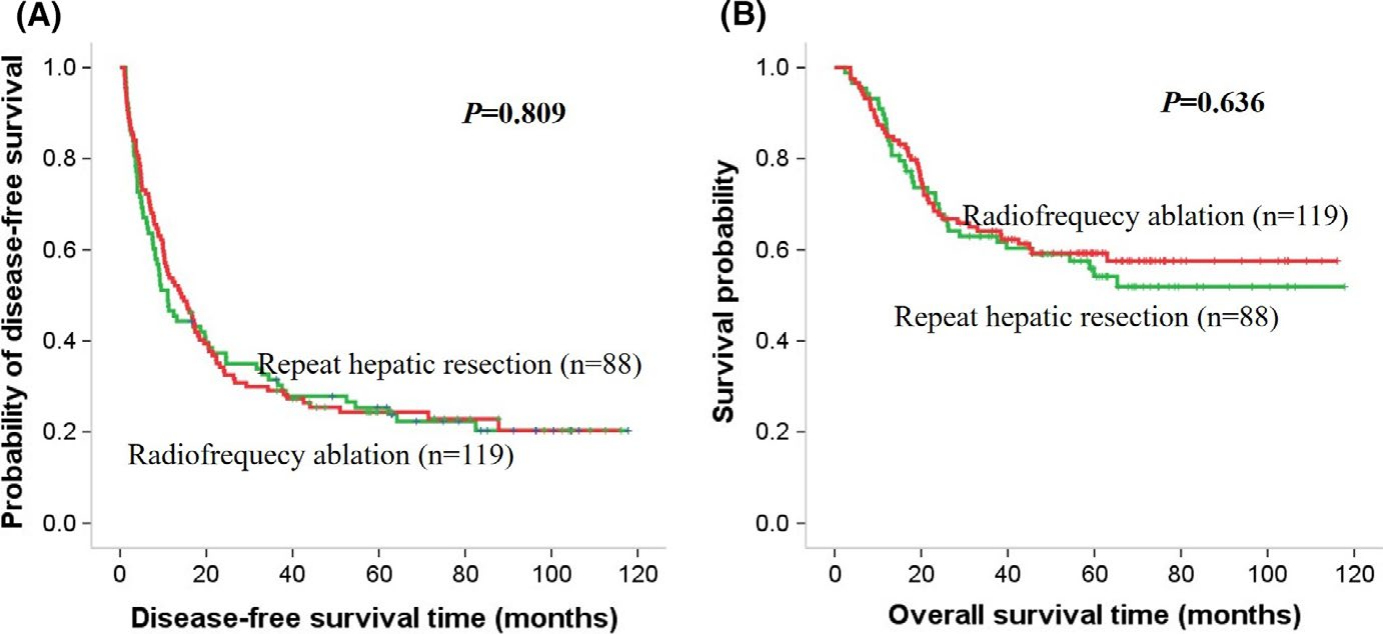
Kaplan–Meier curves of survival outcomes of recurrent early‐stage HCC. Kaplan–Meier curves of (A) DFS and (B) OS in the VETC‐negative cohort

**FIGURE 5:**
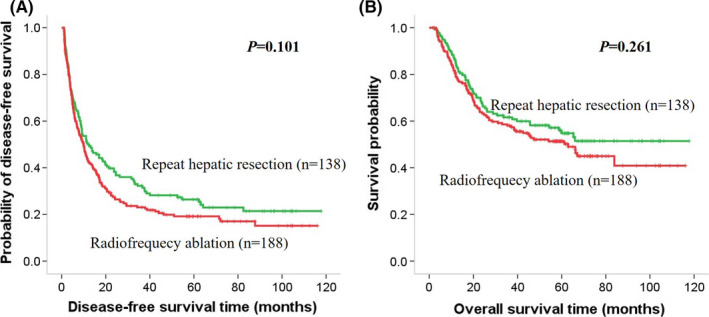
Kaplan–Meier curves of survival outcomes of recurrent early‐stage HCC. Kaplan–Meier curves of (A) DFS and (B) OS in the whole cohort

First, the survival benefit of treatment for recurrent early‐stage HCC in the VETC‐positive and VETC‐negative groups was evaluated. In the VETC‐positive cohort, the 1‐, 3‐, and 5‐year DFS rates were 58.0%, 30.0%, and 14.0% for the RHR group and 26.1%, 8.7%, and 4.3% for the RFA group, respectively. The RHR group had a longer median DFS time compared to the RFA group (15.0 vs. 5.0 months, *p *= 0.001). Similarly, the 1‐, 3‐, and 5‐year OS rates for RHR group and RFA group were 82.0%, 54.0%, and 28.0% and 69.6%, 37.7%, and 13.0%, respectively (*p *= 0.008). The RHR group had a longer median OS time compared to the RFA group (39.5 vs. 19 months, *p *= 0.001) (Figure 3). In contrast, in the VETC‐negative cohort (Figure 4) and the entire cohort (Figure 5), there was no significant difference in DFS and OS rates between the RHR and RFA groups (*p *> 0.05) (Figure 4).

### 3.3 Characteristics after treatment

Treatment‐related complications are summarized in Table S2. Complications were reported according to the Clavien–Dindo classification. No treatment‐related mortality was reported in this study and the incidence of major complications in the RHR group did not differ significantly from that of the RFA group. Nevertheless, the delay in the RHR group resulted in a notably longer hospital stay (6.7 vs. 4.1 day, *p *< 0.001) and higher total hospital charges (52,645 vs. 27,949 RMB, *p *< 0.001).

## 4 DISCUSSION

Because of the high incidence of intrahepatic recurrence, which affects the prognosis of HCC patients, the available treatment options are not that different from those for primary tumors. Many recent articles have reported the feasibility, safety, and efficacy of RHR or RFA for intrahepatic HCC recurrence. However, there is no consensus on the most appropriate choice for HCC recurrence with regard to RHR or RFA. Our study demonstrated that RHR was relatively safe and superior to RFA in improving survival outcomes for the recurrence of early‐stage HCC for our whole cohort. Moreover, we first explored the VETC pattern to identify the patient subgroup most likely to benefit from this treatment. The result showed that HCC patients who were VETC‐positive could benefit from RHR, while there was no significant difference between RHR and RFA in the VETC‐negative cohort.

Indeed, following the surgical resection of the primary tumors, intensive screening was usually applied using AFP levels and CT or MRI, and the recurring tumors were usually detected in the early stage, with less than 3 cm tumor size.^26^ Our study showed that approximately 30% of recurrent HCCs were smaller than 3 cm, which was more similar to the real situation in the recurrence of tumors. As expected, recurrent early‐stage HCC with a smaller tumor size was closely related to a higher rate of complete tumor elimination after RFA and a greater safety resection margin with fewer resections of tumor‐free liver parenchyma in RHR, which in turn produces a better prognosis. However, different from the prognosis of first treatment in early‐stage HCC, several studies have shown that 70–80% of patients developed a second recurrence with either RHR or RFA.^27^, ^28^, ^29^ Our study found that 78.5% of patients had recurred and 46.9% had died of tumor progression after the second treatment. This may be because recurrent HCC, with a higher degree of malignancy, more easily leads to a second recurrence after treatment.

The debate about whether resection or ablation should be first‐line therapy for early‐stage HCC has intensified over the years and there has also been controversy about treatment optimization for recurrent early‐stage HCC. Several studies revealed similar survival outcomes between RHR and RFA in recurrent small tumors.^22^, ^30^, ^31^ Compared with RHR, RFA is a highly selective, targeted thermal treatment technique to conserve tumor‐free liver parenchyma and cause a minimum of damage to the small or cirrhotic liver remnant. Unlike surgery, RFA can be performed under conscious sedation and has a shorter hospital day making it more cost‐effective than surgical resection. In our study, the median total hospital stay duration was shorter and median total charges for patients who underwent RFA were significantly lower than those for patients who underwent RHR. Therefore, RFA is usually the first choice for patients with early‐stage HCC, whether primary or recurrent. Our study showed that 57.7% of patients had RFA and 42.3% of patients had RHR. No significant difference in survival outcomes between RFA and RHR in the whole cohort could be observed.

The VETC pattern has been identified as an effective predictor of survival in patients with HCC after resection and it can be easily identified and distinguished from capillary vessels by IHC staining for a single endothelial marker, CD34.^17^ In our study, we found that the VETC pattern was correlated not only with recurrence after the first resection but also with the second recurrence. Furthermore, we found that the VETC‐positive patients in the RHR group had longer median DFS and OS rates than those in the RFA group, while there was no significant difference in DFS or OS rates between the RHR and RFA groups among the VETC‐negative cohort. VETC is closely associated with tumor micrometastases and venous thrombus in the tumor margins. We postulate that RFA cannot achieve sufficient ablation in hepatic tumors with potential adjacent venous invasion or microscopic lesion remnants. In contrast, RHR can usually achieve a relatively satisfactory margin of normal liver tissue if possible. Another explanation may be that RFA is inferior to surgery in local tumor control. Recurrence more than 2 years after initial resection is now commonly considered to attribute to multicentric carcinogenesis due to hepatitis or cirrhosis. As the latest advancement of hepatic surgical techniques, anatomic resection is well developed and is performed more actively for hepatic tumors, which can remove the tumor and the entire Couinaud segment potentially containing the adjacent venous invasion or microscopic lesions simultaneously.^32^

Several limitations existed in our study. First, since this was a retrospective study, selection bias may have influenced the results. Second, treatment selection for recurrent HCC was decided by our multidisciplinary team and not randomly assigned. For HCC patients who had a major resection, when recurrence was present and located deep within the liver or in a patient with insufficient liver function reserve, RFA was usually the first choice as it causes less damage, making patients more likely to benefit from treatment.

## 5 CONCLUSIONS

In conclusion, the results of our study showed that RHR was relatively safe and superior to RFA in improving survival outcomes for recurrent early‐stage HCC after initial hepatectomy. Additionally, the VETC pattern may represent a reliable marker for selecting HCC patients who may benefit from RHR; however, large‐scale studies are required to confirm these findings.

## 6 ETHICS APPROVAL AND CONSENT TO PARTICIPATE

All study participants provided informed consent. The study got the approval of the Institutional Review Board of Sun Yat‐sen University Cancer Center and conducted in line with the ethical guidelines of the Declaration of Helsinki.

## CONFLICT OF INTEREST

None reported.

## Supporting information

Table S1

Table S2

## Data Availability

The dataset analyzed during the current study are available from the corresponding author on reasonable request.
