# Supplementary material for: The predictive value of vessels encapsulating tumor clusters in treatment optimization for recurrent early‐stage hepatocellular carcinoma
Source: Cancer Med. 2021 Jul 1;10(16):5466–74. doi: 10.1002/cam4.4102 (PMC8366089; doi:10.1002/cam4.4102)
Supplement: Supplementary file 1 — Table S1 [file CAM4-10-5466-s002.doc]

| **Supplementary table 1** The relationship between VETC and other parameters | | | | | | |
| --- | --- | --- | --- | --- | --- | --- |
|  | VETC (+) | | | VETC (-) | | |
| Variables |  | |  | | | *P* |
| Age, y | 49.1±10.8 | | 50.3±10.8 | | | 0.339 |
| Sex |  |  |  | |  | 0.321 |
| Male | 111 | | 186 | | |  |
| Female | 8 | | 21 | | |  |
| HBsAg |  |  |  | |  | 0.442 |
| Positive | 102 | | 170 | | |  |
| Negative | 17 | | 37 | | |  |
| Background liver |  |  |  | |  | 0.188 |
| Normal | 48 | | 68 | | |  |
| Cirrhosis | 71 | | 139 | | |  |
| Histological grade |  |  |  | |  | 0.907 |
| Well differentiated | 48 | | 86 | | |  |
| Poorly differentiated | 71 | | 121 | | |  |
| Microvascular invasion |  |  |  | |  | <0.001 |
| Present | 53 | | 38 | | |  |
| Absent | 66 | | 169 | | |  |
| ALB at recurrence, g/L | 42.0±4.9 | | 42.5±6.9 | | | 0.446 |
| TBIL at recurrence, umol/L | 15.1±4.8 | | 15.3±5.8 | | | 0.678 |
| HGB at recurrence, g/L | 143.8±17.2 | | 143.4±23.3 | | | 0.880 |
| AFP at recurrence, ng/ml |  |  |  | |  | 0.222 |
| > 20 | 85 | | 133 | | |  |
| ≤ 20 | 34 | | 74 | | |  |
| Child-Pugh score at recurrence |  |  |  | |  | 0.8833 |
| 5 | 96 | | 167 | | |  |
| 6 | 23 | | 38 | | |  |
| Tumor size at recurrence, cm | 2.3±0.6 | | 2.4±0.5 | | | 0.205 |
| Tumor multiplicity at recurrence |  |  |  | |  | 0.370 |
| Solitary | 101 | | 166 | | |  |
| Multiple | 18 | | 41 | | |  |
| Time to recurrence |  |  |  | |  | <0.001 |
| < 1 year | 50 | | 88 | | |  |
| ≥ 1 year | 69 | | 119 | | |  |
| ECOG-PS |  |  |  | |  | 0.819 |
| 0-1 | 112 | | 192 | | |  |
| 2 | 7 | | 15 | | |  |

Abbreviations: ALB, albumin; TBIL, total bilirubin; HGB, Hemoglobin; AFP, α-fetoprotein; ECOG-PS, Eastern Cooperative Oncology Group Performance Status; RFA, radiofrequency ablation; RHR, repeat hepatic resection; VETC, Vessels Encapsulating Tumor Clusters.
