# Supplementary material for: The predictive value of vessels encapsulating tumor clusters in treatment optimization for recurrent early‐stage hepatocellular carcinoma
Source: Cancer Med. 2021 Jul 1;10(16):5466–74. doi: 10.1002/cam4.4102 (PMC8366089; doi:10.1002/cam4.4102)
Supplement: Supplementary file 2 — Table S2 [file CAM4-10-5466-s001.docx]

| Variables | RHR | RFA | *P* |
| --- | --- | --- | --- |
| Postoperative hemorrhage |  |  | 0.087 |
| Absent | 135 | 187 |  |
| Present | 5 | 1 |  |
| Ascites |  |  | 0.120 |
| Absent | 128 | 182 |  |
| Present | 10 | 6 |  |
| Bile leakage |  |  | 0.178 |
| Absent | 136 | 188 |  |
| Present | 2 | 0 |  |
| Pleural effusion |  |  | 0.178 |
| Absent | 136 | 188 |  |
| Present | 2 | 0 |  |
| Liver failure |  |  | 0.167 |
| Absent | 134 | 187 |  |
| Present | 4 | 1 |  |
| Length of stay (days) | 8.7±1.2 | 4.3±0.9 | <0.001 |
| Total hospital charges (RMB) | 52,645±7055 | 27,949±3165 | <0.001 |

**Supplementary table 2** Major complications and characteristics after treatment

Abbreviations: RFA, radiofrequency ablation; RHR, repeat hepatic resection.
